# Habituation/Fatigue behavior of a synapse memristor based on IGZO–HfO_2_ thin film

**DOI:** 10.1038/s41598-017-09762-5

**Published:** 2017-08-24

**Authors:** Ran Jiang, Pengfei Ma, Zuyin Han, Xianghao Du

**Affiliations:** 1 School of Microelectronics, Shandong University, Jinan, 250100 China; 2 School of Physics, Shandong University, Jinan, 250100 China

## Abstract

A synaptic memristor based on IGZO and oxygen-deficient HfO_2_ films has been demonstrated. The memristor exhibits a fatigue response to a monotonic stimulus of voltage pulses, which is analogous to the habituation behavior of biological memory. The occurrence of habituation is nearly simultaneous with the transition from short-term memory to long-term memory. The movement and redistribution of oxygen species with the assistance of polarization in HfO_2_ layer are responsible for the above results. The observation of habituation behavior proves the potential prospect of memristor on the mimic of biological neuron.

## Introduction

The human brain deals with information in parallel, which can easily recognize objects and visual information in complex environment^1^. Therefore, many efforts have been made to realize the neuromorphic computation. The synapse emulation is a key step to build neuromorphic systems that can mimic the human brain^2^. However, the traditional methods, such as the software-based method by conventional von Neumann computers or the hardware-based method by lots of resistor and capacitors in CMOS integrated circuits, occupy large areas and consume much more energy^3^. Nowadays, the realization of a single device with synaptic functions has attracted much attention for the implementation of the neuromorphic system. Among them, the electronic synapses based on memristors have been widely focused^4–6^. In 1971, Chua predicted the fourth basic circuit element, namely, memristor^7^. Subsequently, many studies demonstrated that a memristor can be used as an electronic synapse with its conductance representing the synaptic weight^8–13^.

Although synaptic operation of memristors has been widely demonstrated, the biological properties of habituation/fatigue behavior were not reported for the memristor. An inorganic memristor is so different with a real organic synaptic due to the absence of biological activity, which leads to suspicion for the possibility of achieving a true bio synaptic device. Encouragingly, the paired-pulse facilitation (PPF) behavior has recently been reported^14^, which shows the resemblance between an inorganic memristor with a biological synapse as in response to electrical stimulation. The mechanism responsible for this PPF behavior was demonstrated to be the overlap effect of the two pulses on the memristor^14^. However, when exposed to continuous stimulus, does a memristor show a monotonous increase, that is similar to that of PPF (only a pairs of pulses) measurement? Meanwhile, it has been widely reported that the accumulation of electrical stimulus can make the transition from short-term memory (STM) to long-term memory (LTM)^15^, what is the correlation between this transition with the accumulation of electrical stimulus? In this work, synaptic memristors were fabricated based on the structure of the over-oxidized IGZO and oxygen-depleted HfO_2_ (OD-HfO_2_) films. The habituation in the case of continuous electrical pulse stimulation is observed, which occurs almost simultaneously with the transition from STM to LTM. This interesting behavior of synaptic memristor under continuous stimulation gives the potential application of the simulation of the biological synaptic with the inorganic memristor.

## Methods

### Flow process of fabrication

The memristor structure was fabricated with the following sequence. First, a 150 nm Al layer was deposited on the SiO_2_/Si film with thermal evaporation. Then, a 50 nm HfO_2_ thin film was deposited on the Al/SiO_2_/Si substrate by radio frequency (rf) magnetron sputtering of a Hf target (>99.99% purity) in a mixed Ar/O_2_ ambient. In order to ensure a low oxygen ratio, the oxygen bias is 8 × 10−5 Torr and the Ar bias is 2 × 10−3 Torr. The Ar/O_2_ ratio has been verified to form oxygen-deficient oxide in the rf sputtering. The rf power used here was 200 W. Then, a IGZO thin film with the thickness of ∼50 nm was deposited onto the HfO_2_ thin film by rf magnetron sputtering of an IGZO target in a mixed Ar/O_2_ ambient with the Ar partial pressure of 2 × 10^−3^  Torr and O_2_ partial pressure of 3 × 10^−4^  Torr. Finally, 100 nm Au/20 nm Ti layer was deposited by electron-beam evaporation at room temperature to form the electrodes of the memristor.

### Characterization with instruments

Electrical characterization of the synaptic memristor was conducted with a Keithley 4200 semiconductor characterization system (including Keithley 3390, 2636 A modes, etc.) at room temperature. Both writing and reading of the memristor were performed in the pulse mode. A flow of current from top to bottom electrode was defined as the positive bias.

## Results and Discussion

**Figure 1:**
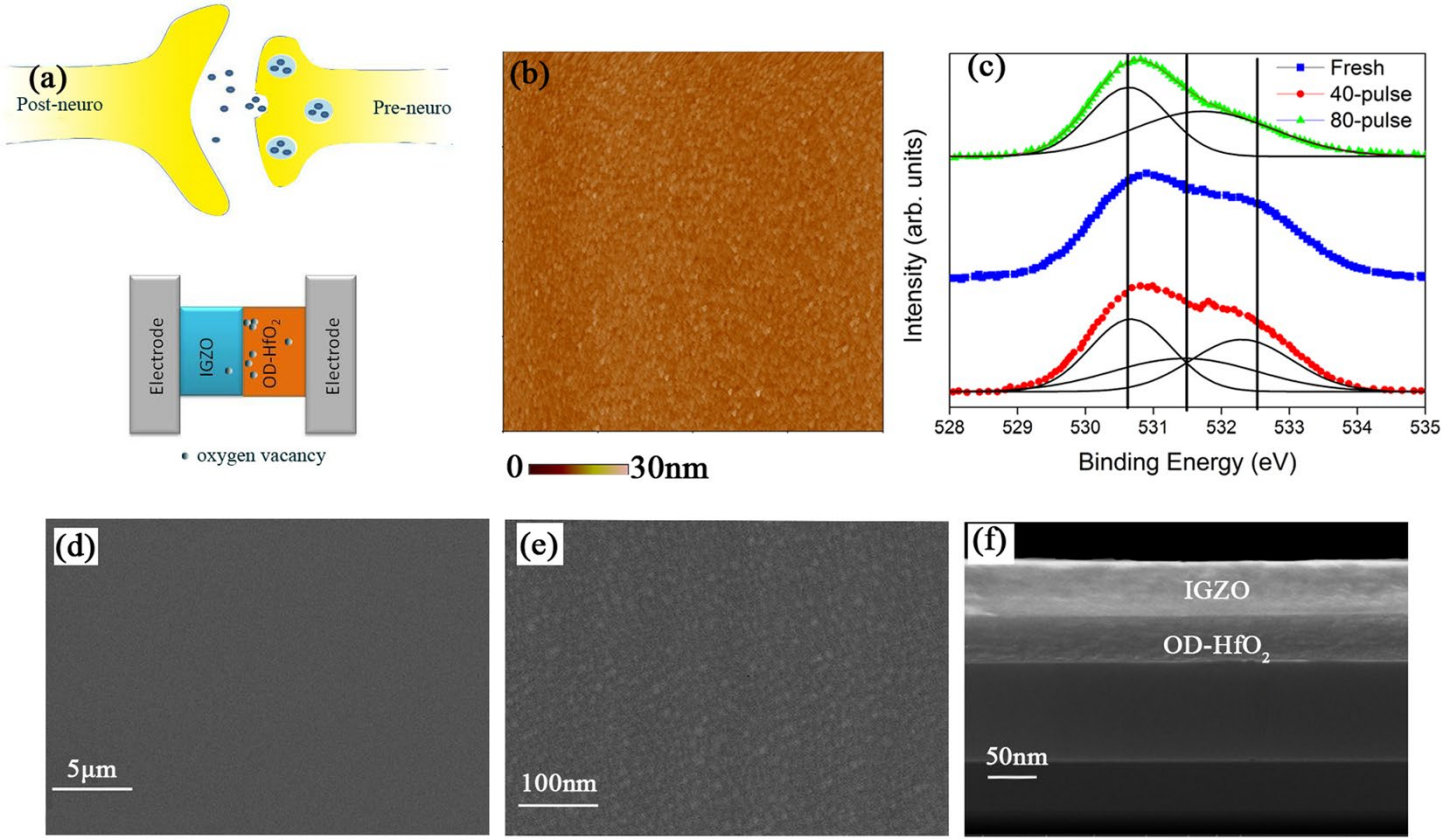
Basic characteristics of fabricated simples. (**a**) Schematic illustration of a biological synapse and the IGZO-HfO_2_-based synaptic memristor. (**b**) AFM morphology of the IGZO surface of a pristine sample. (**c**) XPS spectra for the devce under various pulse number stimulations. (**d**) SEM images of IGZO for the pristine sample. (**e**) SEM images enlarged for 50times. (**f**) The lateral profile for the pristine sample.

The switching layer of the synaptic device consists of two parts: the oxygen-rich IGZO and the oxygen-deficient HfO_2_ layer. The two-terminal, bilayer IGZO–HfO_2_ memristor is analogy to a biological synapse, and oxygen vacancies are similar to neurotransmitters, as shown in Fig. 1(a). The device conductivity was treated as synaptic weight in this memristor. Similar to biological synapse, the synaptic weight can be dynamically modified and stored using consecutive spikes^16^. The release and restored back of oxygen vacancies under the stimulation of the pulses play a role similar to that of a neuro-transmitter in the modulation of the strength of the synaptic connection in a biological synapse^17, 18^. The microstructure and composition of the IGZO films were characterized by SEM and XPS analyses. The films were in high quality and smooth morphology as observed from the AFM and SEM images as shown in Fig. 1. Figure 1(c) shows the XPS spectra for the IGZO film under various pulses stimulations, which will be discussed further below.

**Figure 2:**
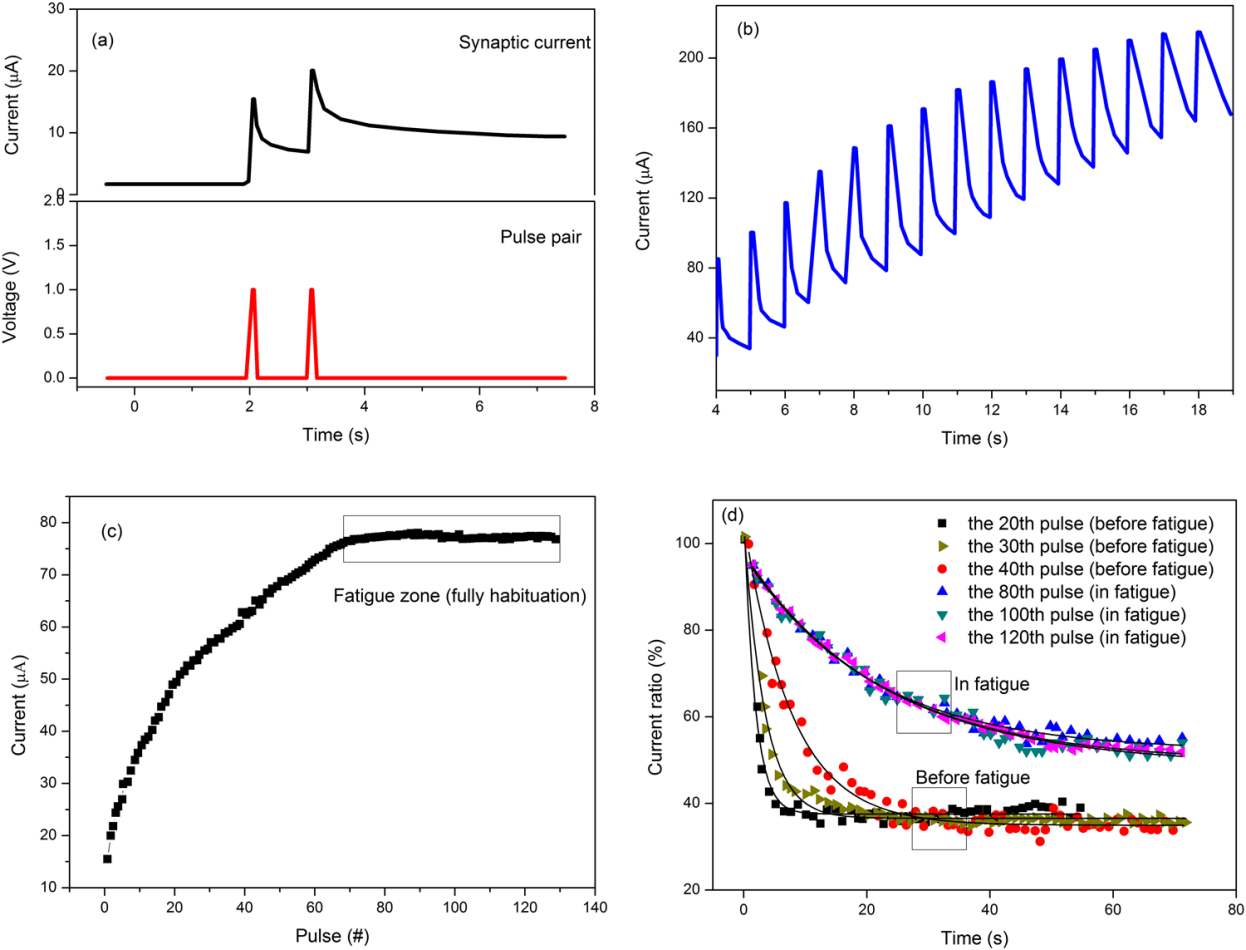
(**a**) Synaptic currents of the memristor triggered by a pair of pluses. The pulse intensity, width, and interval are 1 V, 50 ms, and 1 s, respectively. (**b**) The post synaptic current of the memristor in response to the pulse train. As consecutive voltages are applied, on one hand the device conductivity continuously increases, on the other hand, the magnitude of this increase is gradually weakened. (**c**) The current as a function of the pulse numbers. (**d**) Current decays recorded after different numbers of pulse stimuli. In the fatigue region, the current decay rate is almost the same. the current decay curve was significantly different for different pulse counts for the device unreached fatigue due to insufficient pulse training.

**Figure 3:**
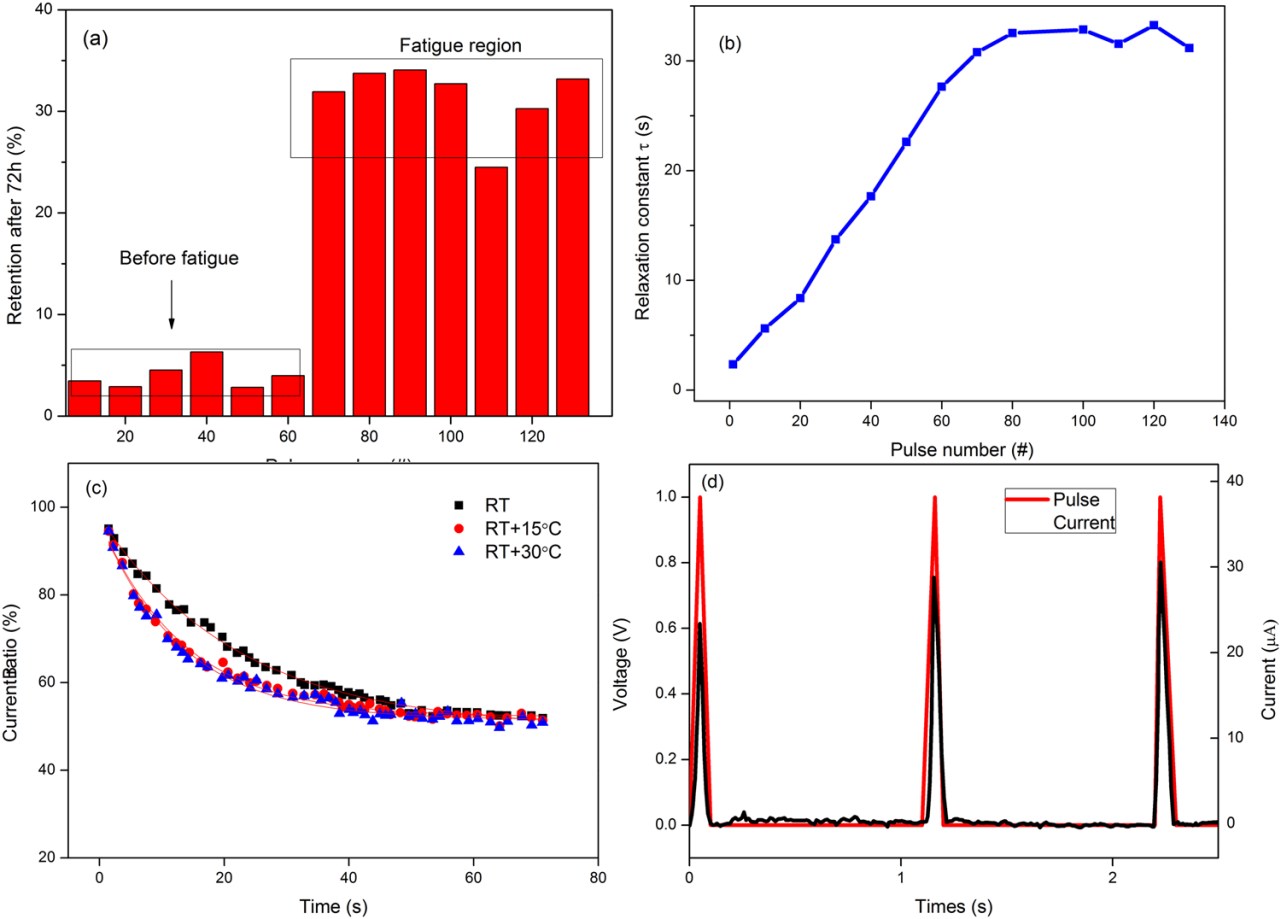
Influence of some parameters on fatigue effect. (**a**) Retention of current decay for the devices with different number of pulse stimulation after 72 h. (**b**) variation of relaxation time constant (*τ*) with the number of stimulation pulses, where the *τ* is obtained by fitting data from Fig. 2(d). It is highly resemble with the Fig. 2(c). (**c**) Current decay for the device stimulated by 120th pulses at different temperatures. Solid lines are fitted curves using Equation 1. (**d**) Current evolution of pure IGZO device (without OD-HfO_2_ layer) for comparison. Current was ceased during the off period of the pulses.

As shown in Fig. 2(a), two successive pulses with fixed intensity and width were applied to the memristor. The postsynaptic current triggered by the second pulse is greater than the first pulse, which is similar to the PPF behavior in the biological synapse. PPF is a plasticity of biological synapse in which spike-induced postsynaptic responses increase when the second spike closely follows the previous spike^19^.When the voltage is turned off, the postsynaptic current does not immediately disappear. In contrast, an attenuation of postsynaptic currents is observed during the off-cycle of the pulse. The attenuation of postsynaptic currents resembles memory loss in biological systems. As is known, PPF is associated with incomplete compensation of oxygen vacancies. After the first pulse, the oxygen vacant filament can be compensated by oxygen, and if the pulse interval is sufficiently small, the oxygen vacancies will not be fully compensated, and therefore the conductive channel will not completely disappear and, after the second pulse, the synaptic current will be greater than the previous one as shown in Fig. 2(a). This kind of PPF behavior stimulated by a pair of pulses in synaptic memristor provides the possibility to study the post-synaptic currents as a function of the number of pulses. As shown in Fig. 2(b), The device conductivity continuously increases as consecutive voltages are applied. An interesting phenomenon is that the current increase magnitude is gradually weakened as the number of pulses increases. In other words, although the stimulus still makes the follow-up current larger than the previous one, the increasing magnitude was gradually weakened, which is similar to the habituation of the brain to frequent boring stimuli. This observation of IGZO-HfO_2_ device shown in Fig. 2(b) expands the facilitation behavior of memristor, since more than two pulses were applied. Figure 2(c) shows the current as a function of the pulse numbers. When the number of the pulses increases, the increasing magnitude of each current decrease gradually. After a certain number, the current nearly keeps constant. The current decay processes were also compared for the devices under different numbers of stimulation pulses. A phenomenon similar to the brain’s forgetting behavior was discovered as shown in Fig. 2(d) that the forgetting time (the memory decay time from beginning to the saturation) increasingly evolves from several seconds to tens of seconds with increasing number of stimulations, and the corresponding retention ratio increases from around 37% to around 61%, indicating a decreasing forgetting rate. As known, the memory behavior in psychology can be categorized into short-term memory (STM) and long-term memory (LTM) based on the retention time^15^. The transition from STM to LTM can be realized by repeating the pulse stimulus, which is analogous to the rehearsal of the biological brain. In this work, the memristor fatigue achieved through repeated stimulation shows a clear synchronization indication of the STM-to-LTM transition, since the current curves in fatigue region were almost unchanged as shown in Fig. 2(d). Contrarily, the current has an obvious rapid-decay zone as the pulse stimulation does not make the memristor reach the “fatigue” region, as shown the 20^th^ and 40^th^ pulses in Fig. 2(d). Clear divide for the current curves shown in Fig. 2(d) was observed, which is synchronized with the device habituation/fatigue under sufficient stimulation. STM and LTM are difficult to define from a biological point of view, and the biological definition of STM and LTM are defined usually as follows: The STM (LTM) is a temporary (permanent) potentiation of neural connections, and lasts for a few minutes or less (from hours to years). Besides, STM can be converted to LTM through repeated rehearsals, which involves a physical change in the structure of neurons. Here, one can find the similarity of this definition to our devices, since these temporal characteristics of memory retention were also achieved in the current device. For devices that did not reach the “fatigue region”, the current ratio of ~55% only lasted for less than 20 seconds, whereas for “fatigue” ones, a current ratio of ~55% could reach a time of not less than 70 s (longer time can also be achieved, as shown in Fig. 3(a)). The 55% ratio was selected because that the current decays into fatigue saturation at approximately this value. The critical threshold is almost synchronized with the occurrence of fatigue behavior (approximately 60–65 pulses, as shown in Figs. 2(c) and Figure 3(a)). Therefore, once the memristor reaches the “fatigue” zone, the current decay gently and slowly, and the devices were almost unchanged for the current decay characteristics (for example the 80^th^, 100^th^ and 120^th^ pulses, as shown in Fig. 2(d)), such invariance could be an indication of reaching the LTM state.

To quantitatively study the STM behavior, the exponential function including decay term was usually used^20, 21^, as described below to explain the relaxation process

1 $$R(t)={R}_{s}+({R}_{0}-{R}_{s})\exp (-t/\tau )$$

*R*(*t*), *R*_0_, and *R*_s_ are the retention level of current at time *t*, *t* = 0, and saturation state shown in Fig. 2(d). τ defined as the relaxation time which can evaluate the forgetting rate of memristor. The current retention level (*R*_s_) of LTM should remain stable for a long time, defined as 72 h here as shown in Fig. 3(a). The relaxation processes after different numbers of stimulation pulses were applied to the device as shown in Fig. 2(b) to Fig. 2(d). The results indicate that the forgetting rate can decrease and the ratio of current retention can increase through repeated stimulation. In Fig. 3(b), the derived relaxation time was plotted with pulse numbers. One can find the same saturation characteristic between this time parameter with that of current evolution in Fig. 2(c), which confirms that the saturation of current retention (fatigue response) could be an indication of LTM. Fig. 3(c) presents the decay curves measured at different temperature. The relaxation process of STM was affected by the temperature. The relaxation time decreases as the temperature increases. According to previous literature^22^, the relaxation time of memory current may simplify to be a linear relationship on diffusion coefficient. On the other hand, considering the dynamic movement of oxygen vacancies (ions), the diffusion coefficient *D* shows the function of T in the follow:

2 $$D(T)\propto \exp \,(-E/kT)$$

where *E* represents an activation energy and *k* is the Boltzmann constant. Therefore, the solution of the dynamic diffusion of oxygen vacancy (or charged ions) can be expressed with the equation has the same exponential form as the above decay equation (Equation 1) used to fit the relaxation process in Fig. 2(d), suggesting that the dynamic movement of oxygen vacancies (or charged ions) may be responsible for the transition of STM to LTM.

It is noted that the device structure used here, i.e., IGZO-HfO_2_, is substantially different with the reported ones (basically pure single layer) showing PPF behaviors^15, 23^. It is also interesting that the post-synaptic current persisted upon the cease of the input voltage spikes here, and the current facilitation was more pronounced than previous reported PPF including current keep increasing with a train of pulses (more than two pulses). We thought the highly oxygen deficient HfO_2_ layer plays an important role in these functions. Figure 3(d) shows the current evolution of pure IGZO device (the thickness of IGZO is in the same with the device used above) for comparison. Current was ceased during the off period of the pulses, which is consistent with previous reports. HfO_2_ with different oxygen (oxygen vacancy) content has ever been shown the surprising properties such as p-type conductivity^24^, luminescence^25^, ferroelectricity^26, 27^, intrinsic d^0^ magnetism^28^, excellent buffer function^29^ and so on. Actually, we have also published several works about the ferroelectric behavior of highly oxygen-deficient HfO_2_ dielectric films (OD-HfO_2_) recently^30, 31^. Highly oxygen-deficient state and/or dopant in HfO_2_ films are the primary condition responsible for the formation of the ferroelectrical polarization, since this condition easily leads to lattice distortion to form ferroelectricity^32^. The mechanism of the ferroelectricity of HfO_2_ dielectric is still unclear since its first report in 2011, and many works are on it to date^33, 34^. However, this ferroelectric fact makes some interesting results and shows promising prospect in this work, considering the mature compatibility of HfO_2_ to semiconductor industry process. To the best of our knowledge, the reported electronic synapses were usually stimulated with external electrical stimulus; and few artificial synapses based on self-ferroelectric stimulus have been reported. This leads to the interesting phenomena that the post-synaptic current may be persisted upon the cease of the input voltage spikes.

**Figure 4:**
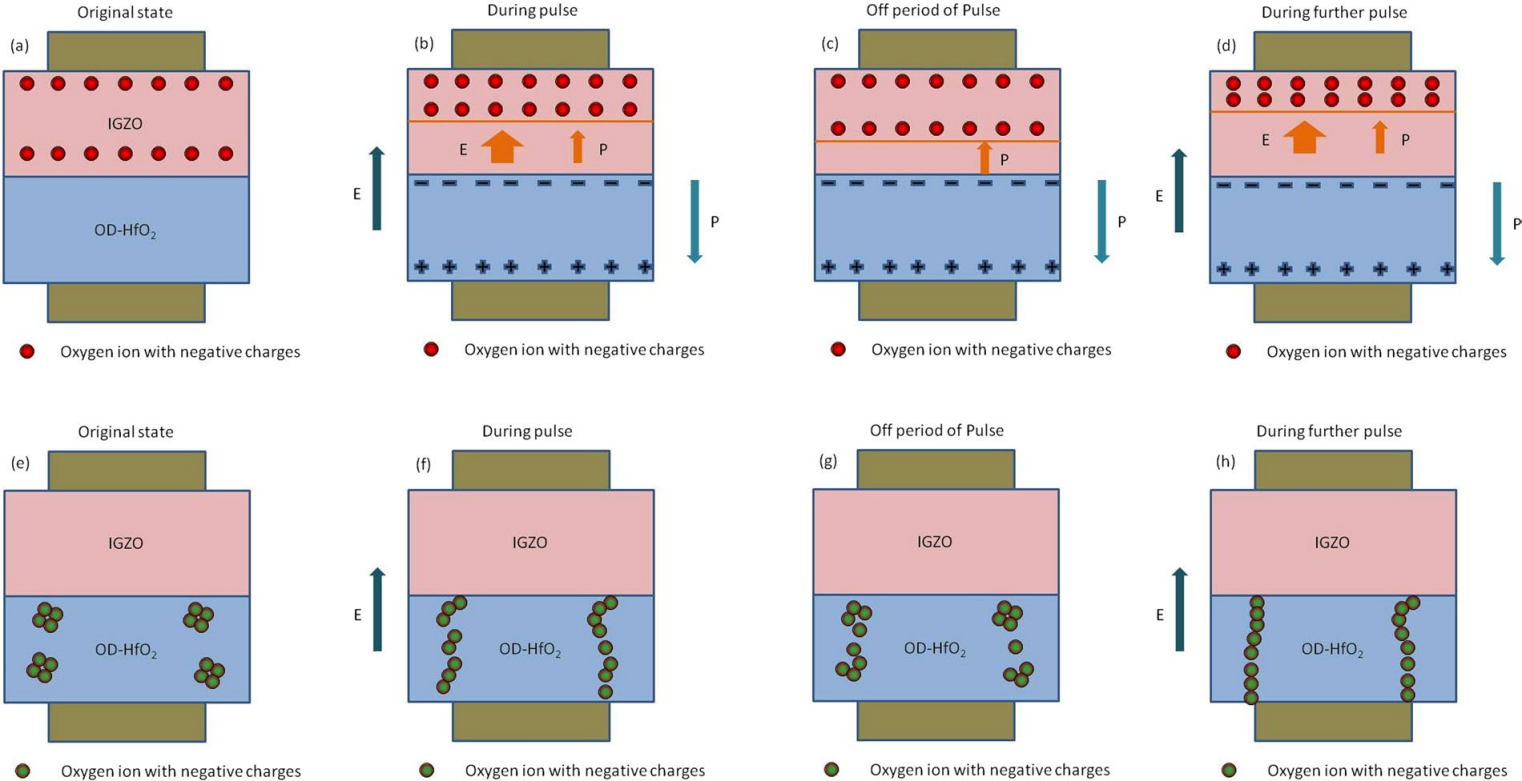
Diagram for the dynamic process for the devices. IGZO layer (**a**) in original state (**b**) during pulse, (**c**) during off period of pulse and (**d**) during further pulse. And HfO_2_ layer (**e**) in original state (**f**) during pulse, (**g**) during off period of pulse and (**h**) during further pulse.

The oxygen ion migration can lead to the concentration differences of the oxygen distribution in IGZO layer, and another dynamic process, ferroelectric-induced ion diffusion was also non-negligible. Considering the migration of oxygen vacancy (equivalent to oxygen ions with negative charges, O^2−^), we can understand the memory mechanism as shown in Fig. 4: i) The conductance of IGZO depends strongly on its oxygen content: the higher oxygen content, the lower the conductivity^15^. When a positive bias voltage is applied to the top electrode (oxygen-rich IGZO side), the electric field induced motion of oxygen ions compresses the highly resistive oxygen-rich IGZO layer, thus increasing the device conductivity, and the polarization also began to form in the OD-HfO_2_ layer. The movement of oxygen vacancy induced by electric field (also with the assistance of ferroelectric polarization in OD-HfO_2_ layer) can change the relative thicknesses of oxygen-deficient and oxygen-rich layers, thus modulating the device conductance. The above process was illustrated in Fig. 4(a) and (b). ii) When the bias is removed, the oxygen vacancy (ions) was expected to be back restored. However, the spontaneous polarization of OD-HfO_2_ layer restricts this process, therefore, only a partial retreat of the conduction front could be present, which reducing the device conductivity. Such a dynamic process corresponds to the current decay during cease of pulse. The above process was illustrated in Fig. 4(c). iii) When a further pulse is applied after the former one, polarization still exists in the case of short pulse interval, thus the superposition of the two identical dynamic processes pushed the conduction front more forward, thus resulting in the increase of the current. This process was illustrated in Fig. 4(d). High-rate stimulation, where the idle time between pulses is very short, can suppress the back-diffusion of oxygen vacancy. This may be a possible reason why frequent stimulation can prompt the transition of the STM to LTM, because in this process, the HfO_2_ polarization was also stressed to be a more solidified state. The back restore dynamic makes a fraction of the oxygen ions recombine with oxygen vacancies (V_o_^2+^). This process makes the compensation of local structural change which responsible for the STM. However, in our device, the STM may easily transformed to LTM state due to the existence of spontaneous polarization of OD-HfO_2_ layer, and with its further stabilization for the pulse stimuli, which may lead to the observation of novel nonvolatile “training-memory” behavior in our device. Combination of two film is important that the highly oxygen deficient HfO_2_ thin film provides the environment of the ferroelectric polarization response to external voltage, while the sufficient oxygen content in over-oxidized IGZO gives the sufficient negatively charged ions. The conductive path based on oxygen vacancy induces an increase of the post-synaptic current directly^35^.

In addition, our recent work^36^ highly resonates with that in ref. 37, i.e., the existence of locally accumulated clusters in HfO_2_ film (based on oxygen vacancy, analogy to Ag clusters in Prof. Yang’s work) was confirmed using a series of XPS measurements. Therefore, another dynamic process may not be excluded which leads to the PPF-kind of current increasing in HfO_2_ layer together with the mechanism shown in Fig. 4(e-f). The clusters shrink back and stretch out in high resistance state (HRS) and low resistance state (LRS), respectively. Via connecting/rupture between the neighbor cluster sites, the switching of the resistance was formed. Ag nanoparticle and oxygen vacancy may have similar behavior to some extent in mimic synaptic influx and extrusion of Ca^2+^. When a voltage pulse is applied, the local temperature increases due to Joule heating and the potential is tilted by electric forces acting on oxygen vacancy (or charged oxygen ions) clusters with induced charge, both of which cause larger clusters to break up. As the clusters become more uniformly distributed in the active layer, the resistance drops, the current and temperature increase, and a positive feedback results in the formation of a conductive channel. As soon as the power is turned off, the temperature drops, and the oxygen vacancy start to recover back with the loss of the applied voltage. Eventually, most of the oxygen vacancies have recovered back into clusters, and the high-resistance state is re-established along with the original conductive path distribution almost restored, leading to volatility. The model predicts interesting conductance evolution similar to synaptic behavior when a train of pulses is applied. First, when the initial voltage pulse is applied, electric field-assisted diffusion pumps some of the oxygen vacancies out of the cluster and they start to bridge each other. If the time between pulses is shorter than the restore time of stretched oxygen vacancies, more oxygen vacancies are pushed to form conductive channel which leads to the gradual increase of device conductance, similar to the paired-pulse facilitation (PPF) phenomenon in bio-synapses. As for the reason that device conductance finally reaches a saturated conducting (fatigue region), the reason is, as the electric field pumps more and more oxygen vacancies to form connected conductive path, the number of vacancies in original clusters decreases (where the distribution peak decreases as more and more pulses arrive). As a result, the number of oxygen vacancies available decreases and the increasing of device conductance starts to decay. This dynamic process is directly related with the external field, i. e., the “E” illustrated in Fig. 4, rather than internal ferroelectric characteristic of HfO_2_ layer. And it is not responsible for the current presentation during the gap of the pulses. Therefore, it is not conflict with the diagram explanation. This process could exist at the same time, leading to the PPF-kind of current increasing in HfO_2_ layer together.

**Figure 5:**
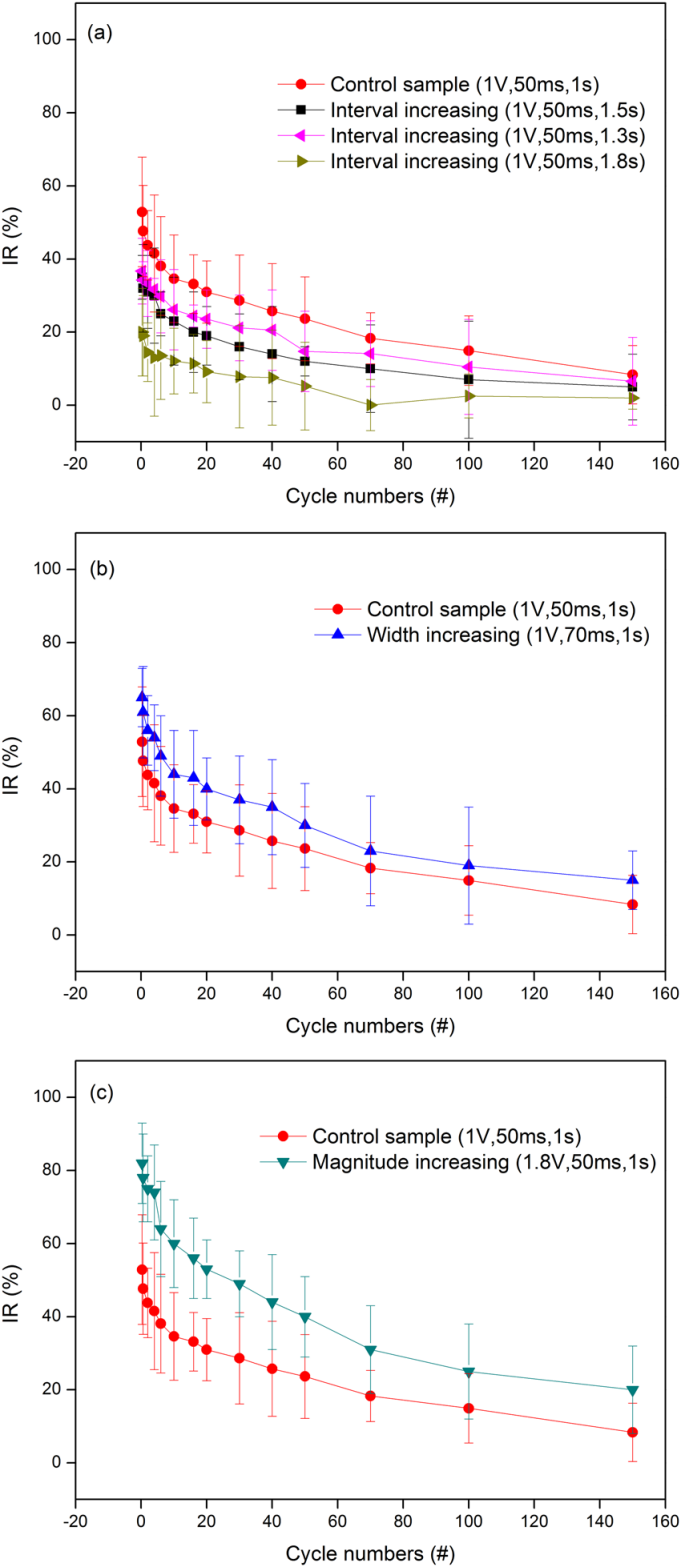
The increasing ratio (IR) versus the cycle numbers for the memristor. (**a**) interval increasing, (**b**) width increasing and (**c**)magnitude increasing.

Generally, the intensity of a pulse is characterized by three parameters: amplitude, interval, and width. In order to investigated the impact of pulse intensity on the increasing magnitude of current, these three parameters were compared as shown in Fig. 5. The ordinate Y (increasing ratio, IR) is defined as follow,

3 $${\rm{IR}}=({{\rm{I}}}_{{\rm{n}}}-{{\rm{I}}}_{n-1})/{{\rm{I}}}_{{\rm{1}}},$$

where I_n_ is the current for the n^th^ cycles. To investigate the impact of each parameter on current increasing ratio, we change one of the parameters of the pulse and fix the remaining two parameters. As shown in Fig. 5, The IR is increased by increasing the intensity of the pulse, i.e. by increasing the pulse width, decreasing the spacing, or increasing the width. However, as the pulse intensity increases, the IR trend is almost constant, i.e., keep decreasing and tend to be saturated. A stronger pulse allows the memristor resistance to be effectively reduced, resulting in a higher current. However, the “fatigue” effect of memristors based on the dynamic mechanism of oxygen vacancies is always present within limited pulse interval, which resembles the memory fatigue of the human brain in repetitive single stimuli. The time interval was varied between the two spikes, and the facilitation is decreased with the interval increase, as shown in Fig. 5. the device conductance increases (PPF) from its initial conductance and interval leads to a reduced rate of increment when the time interval between pulses is long. No further curves shown is because the conductance of the device starts to show no facilitation with longer interval. It is reasonable that sequential pulses with a long enough interval, may form a conducting path first, but before the next pulse arrives the path breaks and the oxygen vacancies (or ions) are re-accumulated back to original position (IGZO) or clusters (HfO_2_). They were previously reported for the cluster distribution (HfO_2_)^36^ and the line forward/back distribution (IGZO)^15^.

In addition, Oxygen ions migration was not only influenced by the electrical filed (drift), but also influenced by the concentration distribution (diffusion). With the charged oxygen ions movement, a barrier in IGZO near the interface region may be decreased/increased with the application/premovement of pulse voltage. The interfacial barrier evolution under the inertial polarization filed and the external voltage field, respectively. Both energy hight and physical width of interfacial barrier were present under these internal and external filed^31, 38^. Figure 1(c) shows the results of the XPS analysis on O 1 s spectrum in a-IGZO film. the 80^th^ XPS spectra was different with the fresh and 40^th^ ones, though the latter spectra were basically consistent. This may a strong evidence for the STM to LTM transition. This indicated the fatigue state (80^th^) truly formed with altered the oxygen bonding and composition in the a-IGZO. Three distinct components of O 1 s peak were fitted by Gaussian Lorentzian deconvolution, which centered at 530.6, 531.5 and 532.6 eV, respectively^38–40^. The binding energy of the spectrum at 531.5 was associated with the oxygen deficient state within the a-IGZO film. The rest two binding energy (530.6 and 532.6 eV) were usually attributed to the presence of stoichiometric and loosely bound oxygen on the surface or interstitial pores of the a-IGZO film. One can find that the subpeak denoted the oxygen deficient state (531.5 eV) was very limited even in the fresh sample, which confirms that the IGZO layer was over oxidized as expectation. Oxygen ions were pushed towards the tops surface under pulse stimulations, then, at fatigue state (80^th^), the surface chemical bonding may structurally change leading to the LTM formation, as shown in Fig. 1(c). It could be observed that the oxygen deficient state was vanished for the subpeak of 531.5 eV absent.

In conclusion, Habituation/fatigue response to pulse stimulations, resembling memory functions of biological systems, have been demonstrated in the IGZO/HfO_2_ memristor. The electrical conductivity increased with the stimulation of continuous pulse, but the degree of increase gradually decreased. Temperature dependence was observed for the relaxation processes of current retention.Dynamic of oxygen vacancy (ions) under external pulse and internal polarization fied is a dominant mechanism of memory evolution in STM. The STM could be enhanced and transferred to LTM by repetitive stimulation training, which is thought to be related to the local structure transition from unstable to solidified with the assistance of pulse train. Though further investigation is keep going to make the synaptic simulation more accurate and comprehensive, the observation of habitual behavior demonstrates the potential of memristors for biological neuron mimics.
